# Differences in the Control of Secondary Peristalsis in the Human Esophagus: Influence of the 5-HT_4_ Receptor versus the TRPV1 Receptor

**DOI:** 10.1371/journal.pone.0159452

**Published:** 2016-07-20

**Authors:** Chih-Hsun Yi, Wei-Yi Lei, Jui-Sheng Hung, Tso-Tsai Liu, William C. Orr, Pace Fabio, Chien-Lin Chen

**Affiliations:** 1 Department of Medicine, Hualien Tzu Chi Hospital, Buddhist Tzu Chi Medical Foundation and Tzu Chi University, Hualien, Taiwan; 2 Lynn Institute for Healthcare Research, University of Oklahoma Health Sciences Center, Oklahoma City, OK, United States of America; 3 Division of Gastroenterology, Department of Clinical Sciences, L. Sacco University Hospital, Milano, Italy; Indiana University School of Medicine, UNITED STATES

## Abstract

**Objective:**

Acute administration of 5-hydroxytryptamine4 (5-HT_4_) receptor agonist, mosapride or esophageal infusion of the transient receptor potential vanilloid receptor-1 (TRPV_1_) agonist capsaicin promotes secondary peristalsis. We aimed to investigate whether acute esophageal instillation of capsaicin-containing red pepper sauce or administration of mosapride has different effects on the physiological characteristics of secondary peristalsis.

**Methods:**

Secondary peristalsis was induced with mid-esophageal air injections in 14 healthy subjects. We compared the effects on secondary peristalsis subsequent to capsaicin-containing red pepper sauce (pure capsaicin, 0.84 mg) or 40 mg oral mosapride.

**Results:**

The threshold volume for generating secondary peristalsis during slow air distensions was significantly decreased with capsaicin infusion compared to mosapride (11.6 ± 1.0 vs. 14.1 ± 0.8 mL, P = 0.02). The threshold volume required to produce secondary peristalsis during rapid air distension was also significantly decreased with capsaicin infusion (4.6 ± 0.5 vs. 5.2 ± 0.6 mL, P = 0.02). Secondary peristalsis was noted more frequently in response to rapid air distension after capsaicin infusion than mosapride (80% [60–100%] vs. 65% [5–100%], P = 0.04). Infusion of capsaicin or mosapride administration didn’t change any parameters of primary or secondary peristalsis.

**Conclusions:**

Esophageal infusion with capsaicin-containing red pepper sauce suspension does create greater mechanosensitivity as measured by secondary peristalsis than 5-HT_4_ receptor agonist mosapride. Capsaicin-sensitive afferents appear to be more involved in the sensory modulation of distension-induced secondary peristalsis.

## Introduction

Primary peristalsis is initiated by a swallow and is the main source of esophageal transit whereas distension-induced or secondary peristalsis functions to maintain an empty esophagus by clearing refluxate from the stomach [1] in the absence of swallowing. Distension-induced secondary peristalsis has been extensively studied regarding its physiological characteristics and clinical applications in a variety of esophageal symptoms and disorders such as non-obstructive dysphagia and gastroesophageal reflux disease (GERD) [2–6]. Secondary peristalsis can be physiologically triggered by different intraesophageal stimuli including air, mechanical distention, or water infusion [4]. The control of secondary peristalsis in the striated muscle is modulated by central mechanisms with vagal afferents, which subsequently results in sequential vagal efferent discharge to the striated musculature of the proximal esophagus [7], while secondary peristalsis in the smooth muscle part is controlled by an intrinsic neuromuscular reflex. Specifically, the intrinsic reflex that modulates secondary peristalsis includes mucosal and intramuscular mechanoreceptors which contribute to initiate and propagate peristalsis [8, 9].

Several types of acid—sensitive ion channels of the esophagus are demonstrated in sensory afferent neurons including acid sensing ion channels, acid-sensitive tandem-pore channels, transient receptor potential cation channels (TRP channels), and ionotrophic purinoreceptor channels [10] Capsaicin as well as acid pH can stimulate the transient receptor potential vanilloid receptor 1 (TRPV_1_) to mediate heartburn sensation [11] and modulate gastrointestinal motility [12, 13]. Previous studies have demonstrated that secondary peristaltic activity and distension sensitivity can be enhanced by esophageal infusion with capsaicin-containing red pepper sauce [14]. Subsequent work has shown that continued exposure of the esophagus to capsaicin infusion is characterized by the activation of TRPV_1_ receptors and then desensitization of secondary peristalsis [15].

Mosapride, a prokinetic agent, stimulates gastrointestinal motility by facilitating the release of acetylcholine from postganglionic nerve endings of the myenteric plexus via 5-hydroxytryptamine_4_ (5-HT_4_) receptors [16]. It has been shown to elevate resting lower esophageal sphincter (LES) pressure [17], and improve esophageal peristaltic function in patients with GERD [18]. In addition, mosapride is further shown to improve esophageal bolus transit in healthy volunteers [19]. Recently, we have shown that mosapride enhances mechanosensitivity to distension-induced secondary peristalsis and facilitates secondary peristaltic contractility, suggesting that the 5-HT_4_ receptors may participate in the modulation of secondary peristalsis [20].

Given the well-established data showing that both capsaicin-containing red pepper sauce and 5-HT_4_ receptor agonist mosapride can enhance esophageal distension threshold and relevant esophageal contractility [14, 20], it is yet to be determined whether those agents have differential effects on secondary esophageal peristalsis. The aim of this study was to investigate the hypothesis that acute application of capsaicin-containing red pepper sauce or the 5-HT_4_ receptor agonist mosapride may have different physiological effects on esophageal secondary peristalsis as induced by slow and rapid air injections.

## Materials and Methods

### Subjects

Fourteen healthy adults (5 women, mean age 22, range 19–26 years) were enrolled into this study by university advertisement. None of them had any esophageal symptoms, or a history of gastrointestinal disease, or drug allergies. None were taking prokinetic medications or reported frequent consumption of red chili pepper. Written informed consent was obtained from each subject and the study was approved by the Ethics Committee of Hualien Tzu Chi Hospital, Taiwan.

### Esophageal manometry

Recordings of intraluminal pressures were made using a Koenigsberg 4-channel 4.5-mm solid-state probe (Sandhill Scientific, Inc., Highlands Ranch, CO, USA) incorporated with one circumferential pressure sensor at 5 cm and three unidirectional pressure sensors at 10, 20, and 25 cm from the tip. The infusion port was in the mid esophagus with its location between 15 and 20 cm from the tip. After an overnight fast, each subject had the catheter passed through the nose in the supine position into the esophagus. By using stationary pull-through technique, the catheter was finally placed with its most distal sensor in the high-pressure zone of the LES. Recordings of intraluminal pressures were continuously stored on the computer for subsequent analysis. We detected swallowing events by the most proximal channel of the catheter, which was located in the pharynx in order to differentiate primary and secondary peristalses.

### Study design

Primary and secondary peristalsis were compared 10 minutes (min) after the infusion of capsaicin-containing red pepper sauce (pure capsaicin, 0.84 mg) or 60 min after oral mosapride (40 mg) [21]. Subjects were studied on two occasions at least 1-week apart. The test capsaicin solution contained 5 mL of red pepper sauce (Tabasco, McIIhenny Company, Avery Island, LA, USA), diluted with 15 mL of saline, which was infused into the mid esophagus via the manometric catheter at a rate of 10 mL/min. The capsaicin dose was based on prior study on secondary peristaslsis in which the total amount of pure capsaicin included in the infused red pepper sauce suspension (5 mL of Tabasco) was equivalent to 0.84 mg of pure capsaicin [13, 14]. The dose of oral mosapride was chosen according to our previous work on secondary peristalsis [20].

Primary peristalsis was tested with ten, 5 mL normal saline swallows. Each swallow was separated by an interval of 20 seconds (sec). Secondary peristalsis was evoked by direct esophagus injection of air accomplished first by a slow air (0.25 mL/sec) via an automatic infusion pump attached to the manometry catheter. The amount of total volume tested was determined according to the rate and time for injection of air for triggering secondary peristalsis. Secondary peristalsis was then triggered with rapid air injection by hand, in which we started at 1-mL volume and gradually increased the volume at 1-mL increments until the response of a secondary peristalsis was seen or injected volume reached 20 mL without a positive response. We determined the threshold volumes for slow and rapid air injections as the lowest injection volume that triggered a secondary peristalsis [22]. Then, ten 20-mL air was injected within 0.5 sec to assess secondary peristaltic response and manometric parameters. Successful response was recognized within a period of 20 sec, during which each subject was instructed not to swallow. At the end of the 20 sec period, each subject was asked to perform a dry swallow that helped clear any residual air before the next stimulus and reduce the desire to swallow.

### Data analysis

Complete primary peristalsis was identified when the pressure wave in the proximal esophagus was greater than 12 mmHg and that in the distal esophagus was greater than 25 mmHg with normal propagation [4]. Peristaltic progression was defined when the minimal latency of wave onset was 0.5 sec between two recording channels. Failed peristalsis was noted when there was a lack of a pressure wave, ≧ 12 mmHg in the proximal esophagus or ≧ 25 mmHg in the distal esophagus, or occurrence of synchronous pressure waves. Secondary peristalsis was determined to be complete according to the criteria given for primary peristalsis [4]. Data for esophageal wave amplitude (mmHg) and duration (sec) of secondary peristalsis were recorded and compared for the distal esophagus.

### Statistical analysis

The frequencies of successful primary and secondary peristalsis were determined in each subject as a percentage of the number for each stimulus. Differences in the frequencies were expressed as median with interquartile range in parentheses and compared using the Wilcoxon signed-rank test. All other data including threshold volumes, peristaltic wave amplitudes, and contractile duration were expressed with mean ± SEM and compared with a paired t-test between capsaicin infusion and oral administration of mosapride. Differences were considered statistically significant when a P-value of < 0.05. Statistical analyses were performed with SPSS 19 for Windows (SPSS, Inc, IL, USA).

## Results

### Comparison of esophageal distension threshold between capsaicin infusion and mosapride administration

**Fig 1:**
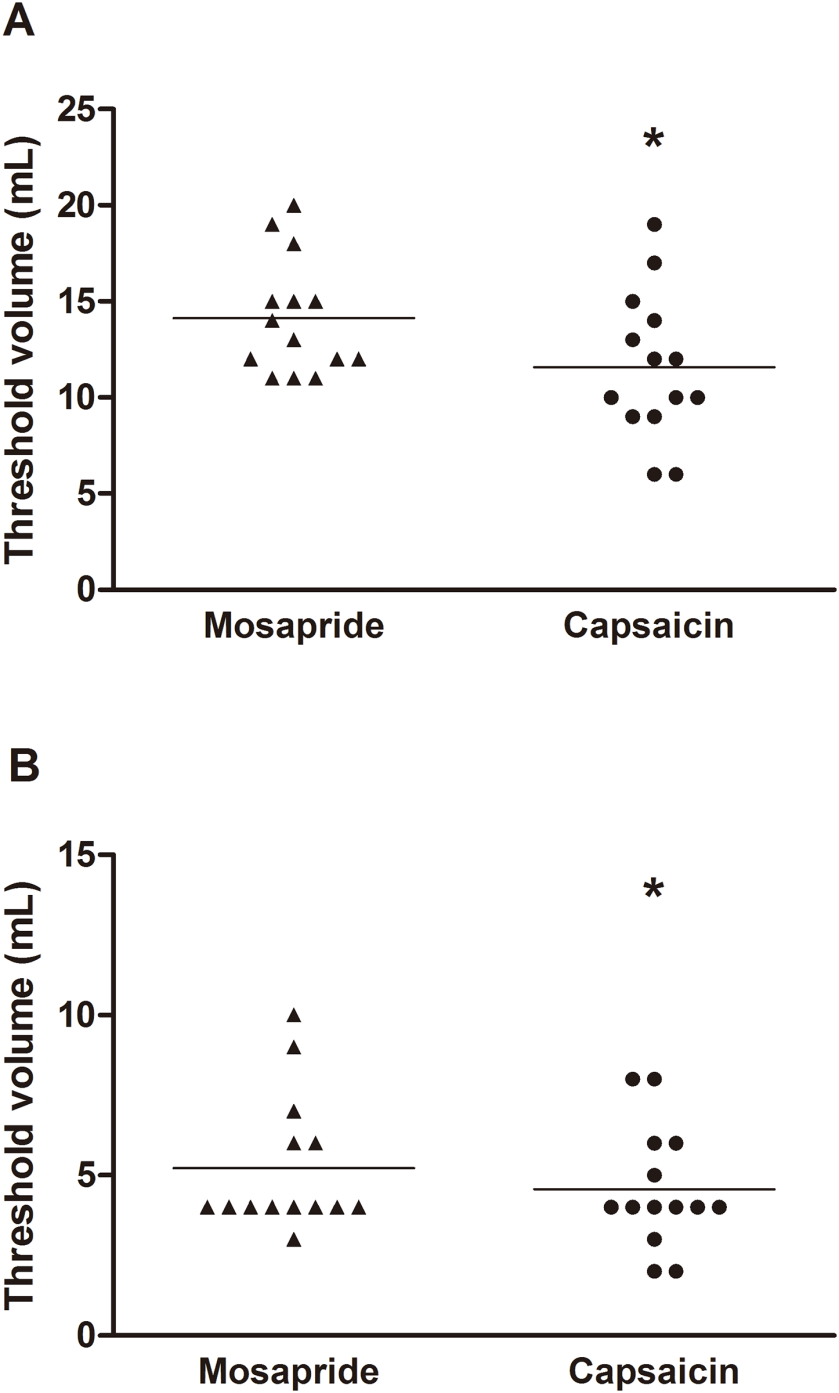
A The threshold volume for triggering secondary peristalsis during slow air injection is significantly lower with capsaicin infusion than oral administration of mosapride (11.6 ± 1.0 vs. 14.1 ± 0.8 mL, *P = 0.02); B the threshold volume for generating secondary peristalsis during rapid air injection is significantly lower with capsaicin infusion than oral administration of mosapride (4.6 ± 0.5 vs. 5.2 ± 0.6 mL, *P = 0.02). Values are expressed as mean ± SEM. Line represents the mean value.

**Fig 2:**
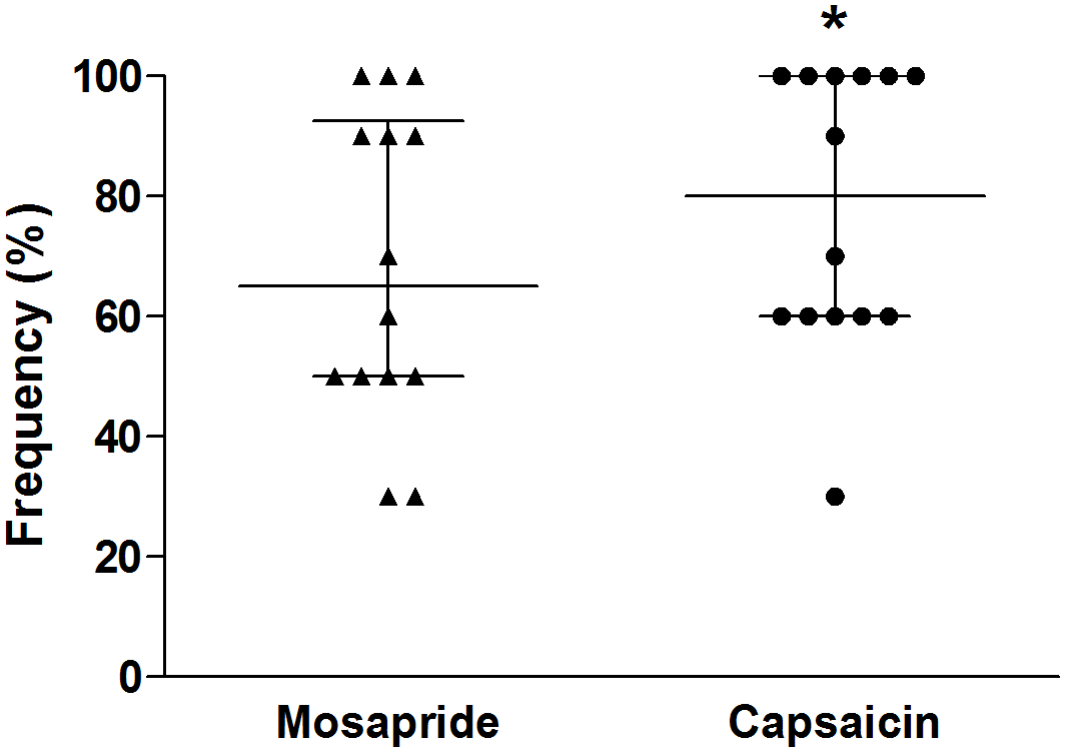
Secondary peristalsis is trigged more frequently in response to rapid air injection with capsaicin infusion than oral administration of mosapride (80% [60–100%] vs. 65% [50–100%], *P = 0.04). Values are expressed as median with interquartile range.

The threshold volume for triggering secondary peristalsis during slow air injection was significantly decreased with capsaicin infusion compared to mosapride (11.6 ± 1.0 vs. 14.1 ± 0.8 mL, P = 0.02) (Fig 1). Similarly, the threshold volume for generating secondary peristalsis during rapid air injection was significantly decreased with capsaicin infusion (4.6 ± 0.5 vs. 5.2 ± 0.6 mL, P = 0.02) (Fig 1). Secondary peristalsis was detected more frequently in response to rapid air injection with capsaicin infusion compared to mosapride (80% [60–100%] vs. 65% [50–100%], P = 0.04) (Fig 2).

### Comparison of primary and secondary peristaltic activity between capsaicin infusion and mosapride administration

Table 1 summarizes the results for primary peristalsis between capsaicin infusion and oral administration of mosapride. No significant difference was found in any of the primary peristaltic parameters or LES pressure between capsaicin infusion and use of mosapride (Table 1). The frequency of primary peristalsis was above 90% for both capsaicin infusion and mosapride (Table 1). There was also no difference in pressure wave amplitude or peristaltic duration during slow or rapid air injection between capsaicin infusion and mosapride (Table 2).

**Table 1:** Effect of mosapride and capsaicin on manometric parameters of primary peristalsis.

	Mosapride	Capsaicin	P-value
**Amplitude of contractions (*mmHg*)**			
Proximal	43.7 (4.8)	42.8 (6.2)	0.8
Middle	80.4 (6.5)	79.9 (8.1)	0.88
Distal	98.6 (8.6)	101.1 (7.5)	0.67
**Duration of contractions (*sec*)**			
Proximal	2.3 (0.1)	2.3 (0.1)	0.4
Middle	2.3 (0.1)	2.3 (0.1)	0.71
Distal	2.6 (0.2)	2.6 (0.1)	0.98
**LES pressure (*mmHg*)**			
Fc Basal	24.0 (3.0)	25.0 (1.9)	0.73
Residual	0.4 (1.3)	1.9 (1.3)	0.23
**Complete peristalsis (*%*)**	90	92	0.57

Values are means (SE) or percentage.

**Table 2:** Effect of mosapride and capsaicin on manometric parameters of secondary peristalsis.

Protocol	Mosapride	Capsaicin	P-value
**Slow injection**			
Peristaltic wave (*mmHg*)			
Eso-m	66.1 (10.0)	65.2 (8.9)	0.64
Eso-d	94.6 (12.8)	87.7 (9.9)	0.66
Duration (*sec*)			
Eso-m	2.7 (0.3)	3.0 (0.7)	0.95
Eso-d	3.0 (0.4)	3.5 (0.8)	0.64
**Rapid injection**			
Peristaltic wave (*mmHg*)			
Eso-m	71.2 (8.5)	73.8 (9.0)	0.72
Eso-d	93.7 (10.8)	102.0 (9.3)	0.35
Duration (*sec*)			
Eso-m	2.4 (0.1)	2.8 (0.2)	0.12
Eso-d	2.8 (0.2)	3.3 (0.2)	0.29

Values are means (SE). Eso-m = mid-esophagus. Eso-d = distal esophagus.

## Discussion

The important finding from this study is that the infusion of capsaicin-containing red pepper sauce directly into mid-esophagus enhances greater esophageal mechanosensitivity stimulating secondary peristalsis and facilitating more secondary peristaltic activities in response to rapid esophageal distension when compared to oral administration of mosapride. These findings are in line with previous works done in humans which documented the presence of prokinetic effects on primary and secondary peristalsis subsequent to capsaicin infusion [14] and the administration of the 5-HT_4_ agonist mosapride [20].

In an early study [20], the 5-HT_4_ agonist mosapride administration was shown to improve contractility in the esophageal smooth muscle with primary peristalsis. In addition, oral administration of mosapride also promoted secondary peristalsis by enhancing esophageal mechanosensitivity of distension-induced secondary peristalsis along with improving the frequency of complete secondary peristalsis as induced by rapid distension of the esophagus [20]. However, besides its prokinetic effect on motor activity of secondary peristalsis, the influence of the 5-HT_4_ agonist mosapride on sensory modulation of secondary peristalsis appears to be more specific to rapid air distension of the esophagus [20]. Of interest is that similar physiological phenomena have been observed with esophageal infusion with capsaicin-containing red pepper sauce [14], in which capsaicin infusion not only improved primary peristalsis but also secondary peristalsis in response to both slow and rapid distension of the esophagus. Since the response to rapid air distension is mediated by mechanoreceptors of esophageal mucosa and that to slow air distension is mediated by those of the esophageal muscularis [9, 23–25], it appears that capsaicin may play a broader role in the modulation of distension-induced secondary peristalsis than oral administration of mosapride. Therefore, based on our findings, it is conceivable that the infusion of capsaicin-containing red pepper sauce is more effective than mosapride regarding the ability to stimulate secondary peristalsis.

The mechanism through which capsaicin infusion could increase secondary peristaltic activity is mainly by altering membrane permeability to Ca^2+^ in primary afferent neurons [26] with consequent release of numerous stimulatory neuropeptides [27]. Previous studies failed to demonstrate any motor response as induced by capsaicin infusion in patients with severe mucosa injury of the esophagus such as Barrett’s esophagus [28]. This suggests that the integrity of sensory afferent modulation of esophageal peristalsis is important to achieve physiological effectiveness of esophageal motility. When compared with the mechanism of capsaicin infusion on esophageal peristalsis, the 5-HT_4_ agonist mosapride appears to provoke the activation of normal peristaltic reflex by enhancing the release of neurotransmitters from mucosally activated submucosal intrinsic primary afferents, which is crucial for its prokinetic effects on esophageal motility [29]. Specifically, the mechanism for its role in promoting esophageal peristalsis is due to the fact that mosapride is reported to act through the release of acetylcholine from postganglionic nerve endings of the myenteric plexus within gastrointestinal smooth muscles [30]. The increase in motor activity is probably due to 5-HT_4_ receptors located on the nerve terminals on myenteric ganglia [31].

We found that capsaicin infusion is more effective than mosapride in sensitizing distension thresholds of secondary peristalsis as triggered by both direct slow and rapid injections of air into the esophagus. Earlier studies have shown that capsaicin has differential effects on esophageal mechanoreceptors [32] with more influence on mucosal rather than muscular mechanoreceptors [33]. This is further supported by other works with repeated esophageal capsaicin infusion in that selective attenuation of secondary peristalsis occurred predominantly with rapid distension, i.e. via rapidly adapting mucosal mechanoreceptors [9], suggesting that distension-induced reflex can be mediated by different type of esophageal mechanoreceptors. However, our prior work demonstrated that secondary peristalsis as induced by slow and rapid air injections can be similarly influenced by capsaicin infusion. Moreover, repeated capsaicin infusion also non-selectively desensitized distension thresholds of secondary peristalsis as induced by slow and rapid air injections after esophageal pretreatment with capsaicin [15]. In contrast, the effect of mosapride on secondary peristalsis only occurred with rapid air injections [20]. These finding may explain why capsaicin infusion appears to be more effective than mosapride in sensory modulation of secondary peristalsis. In addition, it is possible that a central control mechanism may play a role for modulating secondary peristalsis in intact human esophagus when interacting with 5-HT_4_ or TRPV_1_ receptors [34].

In the present study, there were no differences in any of the secondary peristaltic wave amplitudes between capsaicin infusion and mosapride administration, although prior works have shown that both capsaicin infusion and mosapride significantly increased esophageal wave amplitudes of secondary peristalsis [14, 20]. These data suggest that either direct stimulation of mucosal mechanoreceptors or the activation of 5 HT_4_ receptors may similarly activate a reflex mechanism which has been shown to enhance peristaltic amplitude [14, 20]. It is conceivable that while capsaicin [35] stimulates activate C-fiber sensory afferents and consequently enhances esophageal contractility, blood supply, and mucosa integrity by releasing numerous neurotransmitters as well as generating distension-induced reflex [36], mosapride also induces the release of acetylcholine from cholinergic nerve endings and stimulate esophageal motility activity such as secondary peristalsis [20].

There are some clinical implications from these data. It is well established that mosapride helps improve esophageal mechanosensitivity of distension-induced secondary peristalsis and 5-HT_4_ receptors are likely mediators of this response [20] However, current work suggests that capsaicin-containing red pepper sauce creates more potent “prokinetic” influence in the modulation of esophageal reflex in the response to different type of esophageal stimuli in terms of promoting peristaltic efficiency. Furthermore, these findings also extend our prior notion that although esophageal secondary peristalsis triggered by slow and rapid esophageal distension is modulated by TRPV_1_ and 5-HT_4_ receptors, the novel evidence is that TRPV_1_ receptors appear to be physiologically more active in mediating sensory modulation of esophageal secondary peristalsis than 5-HT_4_ receptors during esophageal distension.

The present study therefore has some limitations regarding technique aspects which need to be addressed. First, although the dose we have used in this work is able to successfully induce sensitization in *vivo* effect of capsaicin in order to compare prokinetic effect of 5-HT_4_ agonist mosapride regarding their influences on reflex induced secondary peristalsis it is unknown whether the dosage of either application may have an equivalent effect on mediating the modulation of secondary peristalsis [14, 20]. It may be argued that we didn’t apply median-effective dose or maximum-effective dose, which could be more accurate than what we used in this work. However, in human studies it is somewhat difficult to perform the experiment by using median-effective dose or maximum-effective dose because of potential side/adverse effects that may occur with maximum-effective dose such as capsaicin or mosapride. Finally, the data were obtained using conventional manometry which may be less comprehensive and informative when compared with high resolution manometry [37].

## Conclusions

In conclusion, we confirm the hypothesis that acute administration of capsaicin-containing red pepper sauce suspension does create greater mechanosensitivity in response to slow and rapid distension-induced esophageal reflex (i.e. secondary peristalsis) compared to the 5-HT_4_ receptor agonist mosapride. This study provides additional physiological evidence that in the human esophagus there is evidence for a differential contribution of TRPV_1_ and 5-HT_4_ receptors which mediate the sensory modulation of secondary peristalsis. Capsaicin-sensitive afferents appear to be more involved in sensory control of distension-induced secondary peristalsis in human esophagus, while the motility aspects of secondary peristalsis are equally mediated by both types of receptors.
